# On Subcutaneous Injection of Morphia in Cholera

**Published:** 1863-08

**Authors:** Isaac Ashe

**Affiliations:** Birkenhead


### ON SUBCUTANEOUS INJECTION OF MORPHIA IN CHOLERA.

BY DR. ISAAC ASHE, BIRKENHEAD.

[The following cases occurred in the practice of Dr. Ricketts, of Birkenhead, who, perceiving the value of opium in cholera, and also the difficulty of administering it either by the stomach or rectum, without its being immediately rejected, conceived the idea of employing it as related in sequence, and with success in several instances.]

*Case* 1—Mrs. H——, aged 35, on November 11th, was seized about 7 o'clock A. M., with violent purging and vomiting, occurring about every quarter of an hour, and attended with severe cramps in the bowels, extending down the limbs. The evacuations were of the usual ice-water character; coldness and collapse came on very speedily; and when Dr. Ricketts was sent for at 10 o'clock, or three hours after the commencement of the attack, the patient was already cold and livid in countenance.

Dr. Ricketts immediately injected ♏ xv. of liq. morph. acet. beneath the skin of the abdomen. In quarter of an hour the cramps were completely removed, and the patient expressed herself as very comfortable. Dr. Ricketts administered, also, a few grains of calomel. From the moment of injection there was no return of either purging or vomiting; collapse was removed, warmth returned to the surface, and she rapidly regained strength. The next day she was able to be up, and felt almost as well as ever, being able even to go about and attend to household matters.

*Case* 2—At half-past 12 o'clock the same night, Dr. Ricketts requested me to see a patient, with similar symptoms. He was an old Indian veteran , aged 64,and had often seen men struck down in India with cholera, and was, consequently, very well aware what was the matter of him. I found him suffering from violent purging, which had occurred, he estimated, about twenty times within the eight hours which had elapsed from the commencement of the attack; vomiting had come on about two hours later, and was continuing with great frequency and severity. The cramps in the bowels were so severe, that the stout old soldier was crying out with them; they extended also down the thighs; collapse had not yet commenced, but the surface was much colder than normal, though the pulse was 104. Following Dr. Ricketts' treatment, I injected ♏ xv. of liq. morph. acet. beneath the skin of the abdomen, and, wishing thoroughly to test the efficacy of this plan of treatment, determined to have recourse to no other measures. In a quarter of an hour the cramps were quite gone, and the patient exclaimed about the wonderful powers of the remedy.—They did not again recur, though the purging returned, but with less frequency; vomiting also recurred once or twice during the night. Accordingly, some hours afterwards, Dr. Ricketts injected a second ♏ xv. of the liq., which had the effect of completely arresting all purging and vomiting. The patient was up the next day, complaining only of a little weakness, and on the following day he set out on a journey.—*Med. Times & Gazette, Dec.* 13, 1862, *p.* 644.

